# Impact of the Ketogenic Diet on Linear Growth in Children: A Single-Center Retrospective Analysis of 34 Cases

**DOI:** 10.3390/nu11071442

**Published:** 2019-06-26

**Authors:** Cinzia Ferraris, Monica Guglielmetti, Ludovica Pasca, Valentina De Giorgis, Ottavia Eleonora Ferraro, Ilaria Brambilla, Alessandro Leone, Ramona De Amicis, Simona Bertoli, Pierangelo Veggiotti, Anna Tagliabue

**Affiliations:** 1 Human Nutrition and Eating Disorder Research Center, Department of Public Health, Experimental and Forensic Medicine, University of Pavia, Via Agostino Bassi 21, 27100 Pavia, Italy; 2 Department of Child Neurology and Psychiatry, IRCCS Mondino Foundation, Via Mondino 2, 27100 Pavia, Italy; 3 Unit of Biostatistics and Clinical Epidemiology, Department of Public Health, Experimental and Forensic Medicine, University of Pavia, Via Forlanini 2, 27100 Pavia, Italy; 4 Department of Pediatrics, Fondazione I.R.C.C.S. Policlinico San Matteo, University of Pavia, Viale Camillo Golgi 19, 27100 Pavia, Italy; 5 International Center for the Assessment of Nutritional Status (ICANS), Department of Food Environmental and Nutritional Sciences (DeFENS), University of Milan, Via Sandro Botticelli 21, 20133 Milan, Italy; 6 Pediatric Neurology Unit, Vittore Buzzi Hospital, Via Lodovico Castelvetro 32, 20154 Milan, Italy; 7 Biomedical and Clinical Sciences Department, Luigi Sacco Hospital, University of Milan, via G. B. Grassi 74, 20157 Milan, Italy

**Keywords:** ketogenic diet, growth, drug-resistant epilepsy, glucose transporter type 1 deficiency syndrome, children

## Abstract

Data on the impact of the ketogenic diet (KD) on children’s growth remain controversial. Here, we retrospectively investigated the occurrence of linear growth retardation in 34 children (47% males; age range: 2−17 years) diagnosed with drug-resistant epilepsy (DRE; *n* = 14) or glucose transporter type 1 deficiency syndrome (GLUT1-DS; *n* = 20) who had been treated with the KD for 12 months. The general characteristics of children with and without growth retardation were also compared. All participants received a full-calorie, traditional KD supplemented with vitamins, minerals, and citrate. Most children (80%; 11/14 in the DRE subgroup and 16/20 in the GLUT1-DS subgroup) treated with the KD did not show growth retardation at 12 months. Although participants with and without delay of growth did not differ in terms of baseline clinical characteristics, dietary prescriptions, or supplementation patterns, marked ketosis at 12 months tended to occur more frequently in the latter group. Altogether, our results indicate that growth retardation may occur in a minority of children treated with the KD. However, further research is required to identify children at risk and to clarify how increased ketones levels may affect endocrine pathways regulating growth during KD administration.

## 1. Introduction

The ketogenic diet (KD) is not only considered a valuable non-pharmacological therapeutic option for children with drug-resistant epilepsy (DRE) [1] but it also represents the mainstay of treatment for glucose transporter type 1 deficiency syndrome (GLUT1-DS, OMIM 606777) [2]. Patients with DRE should receive the KD for at least three months, after which it can be continued for several years if symptoms subside (i.e., seizure reduction >50%) [3]. As far as subjects with GLUT1-DS are concerned, the KD should be initiated with the expectation of a lifelong treatment [4].

KD requires constant nutritional monitoring over time both to ensure its effectiveness and to reduce the likelihood of short- and long-term adverse effects [3]. Among the potential long-term consequences, recent years have witnessed a remarkable interest on the possible effects of the KD on children’s growth. Both retrospective [5,6] and prospective [7,8] investigations with follow-up periods up to six months failed to identify a negative impact on growth percentiles. However, numerous long-term studies reported a decrease of height [9] or weight and height [10,11,12,13,14] with the KD. Such discrepancies clearly indicate that this issue deserves further scrutiny.

Data on factors that may affect the growth of children treated with the KD (e.g., ketosis or nutrient adequacy) remain scarce. Peterson et al. [11] demonstrated that subjects with marked ketosis were characterized by a significant decrease in height for age z-scores—an effect which was not observed in presence of moderate ketosis. Spulber et al. [13] confirmed this observation in a sample of 22 children—further reporting a negative correlation between growth rate and blood beta-hydroxybutyrate (β-OHB) concentrations. With regard to nutrient adequacy, Neal et al. [12] failed to identify significant associations between calorie or protein intake per se and the best fit of the line slope for weight, height, or body mass index (BMI). In a retrospective analysis of food diaries collected from 39 children treated with the KD, Nation et al. [6] showed that a caloric and protein intake <80% of the recommended amount and a protein/energy ratio ≤1.4 g protein/100 kcal were associated with a decrease in growth percentile. Notably, urinary ketosis levels were not predictive of children’s growth.

All of these studies have been conducted in patients with DRE. As far as GLUT1-DS is concerned, only one case of a child with a growth deficit related to growth hormone (GH) deficiency has been reported in the published literature [15]. Because patients with GLUT1-DS are expected to require a lifelong assumption of the KD, data on its potential consequences on children’s growth in this clinical population are eagerly awaited. Starting from these premises, we retrospectively investigated the occurrence of linear growth retardation in 34 children diagnosed with DRE (*n* = 14) or GLUT1-DS (*n* = 20) who had been treated with the KD for 12 months. The general characteristics of children with and without growth retardation were also compared. 

## 2. Materials and Methods 

### 2.1. Ethical Approval 

The study protocol complied with the tenets of the Helsinki Declaration and was approved by the ethical committee of the Fondazione IRCCS Policlinico San Matteo of Pavia on 12 November 2018 (code number: 20180083746). All patients and/or caregivers provided their written informed consent.

### 2.2. Patients and Data Collection

Inclusion criteria were as follows: 1) age between 2 and 17 years; 2) diagnosis of DRE (defined as unresponsiveness to at least two anticonvulsant medications) or GLUT1-DS; and 3) treatment with KD for at least 12 months. All participants were consecutively enrolled at the Department of Child Neuropsychiatry of the IRCCS Casimiro Mondino Foundation (Pavia, Italy) between 2008 and 2017. Children with severe organ failure, thyroid disorders, or requiring enteral and parenteral nutrition were excluded. The ambulation status of each child was analyzed in relation to age and physical activity. Data were extracted from a retrospective review of clinical records and entered onto an electronic database. Clinical, anthropometric, growth, and body composition parameters (i.e., weight, length or height, BMI, height velocity, body fat percentage) of the study participants were collected at baseline and at a 12-month follow-up. KD prescriptions and blood β-OHB concentrations were also recorded. 

### 2.3. Ketogenic Diet

Using a standardized approach [16], the Human Nutrition Research Center keto-team implemented a non-fasting dietary protocol—which was uniformly applied in all of the study patients—characterized by gradual increases of the ketogenic ratio. The usual caloric intake as well as food intolerances and preferences were investigated using weekly food diaries. Energy prescriptions were tailored on each patient’s specific requirements (based on basal energy expenditures measured by indirect calorimetry and subsequently corrected for physical activity levels). KD plans with increasing ketogenic ratios were adjusted at the individual level by an experienced dietician. Energy needs were reassessed every 3 months in infants and children up to 10 years and every 6 months in adolescents. As far as macronutrient composition is concerned, a minimum of 0.8−1 g of proteins from animal sources (e.g., eggs, milk, meat, poultry, and fish) were supplied per kilogram of body weight. All of the study participants were prescribed sugar-free multivitamin and mineral supplements according to their age and sex. 

Oral citrates were also administered to prevent the formation of kidney stones. All patients were started at home on a 1:1 ketogenic diet, with ketogenic ratios subsequently increased to 2:1, 3:1, or 4:1 in order to achieve blood β-OHB levels ≥2.0 mmol/L. Family members were instructed to monitor blood β-OHB concentrations twice per day during the induction phase and report values by e-mail to the study investigators. At follow-up visits, each patient underwent 1) measurements of fasting blood ketones, 2) assessments of compliance to the prescribed diet regimen and supplement use, and 3) screening for potential adverse effects. 

### 2.4. Anthropometric, Growth, and Body Composition Parameters

Anthropometric measurements taken by trained dieticians were used to define growth patterns in accordance with standard procedures [17]. Body weight was measured with a digital scale to the nearest 100 g with the subject wearing light clothes and without shoes. The height of children who were able to stand was measured, with shoes removed, to the nearest 1 mm using a stadiometer. Supine length was measured to the nearest 0.5 cm in all children who were unable to stand independently. BMI was calculated as body weight in kilograms divided by height in meters squared. Anthropometric measurements were plotted on standard growth charts (CDC 2000) [18] to calculate weight, height, and BMI percentiles. The reference standards published by the Centers for Disease Control and Prevention [18] were used to determine age- and sex-specific z-scores for weight, height, and BMI. Changes in z-scores occurring between baseline and 12 months were calculated for height (∆ height z-score), weight (∆ weight z-score), and BMI (∆ BMI z-score). 

Height velocity (cm/years) was estimated from height measurements obtained immediately before starting the diet and 12 months thereafter. Reference values for age and sex were obtained from Tanner et al. [19]. In keeping with the methodology proposed by Nation et al. [6], standard growth percentile charts were used to stratify patients into three growth pattern groups after 12 months of KD, as follows: increased (Group-I), tracking (Group-T), and decreased (Group-D) linear growth. Group-I consisted of children who showed an increased linear growth (i.e., crossing of one major percentile of height in an upward fashion). Children with tracking linear growth (i.e., in line with their expected percentile) were included in Group-T. Finally, Group-D consisted of children with a poor linear growth (i.e., crossing of one major percentile of height in a downward fashion). Body composition was measured with bioelectrical impedance analysis using a Handy 3000 device (DS-Medica, Milano, Italy) with the child lying in the supine position. Electrode detectors were applied to the wrist and ankle. Percent fat mass (% FM) was estimated from raw impedance values using the equation of Houtkooper et al. [20].

### 2.5. Statistical Analysis 

Continuous variables were expressed as medians and interquartile ranges (IQRs) and compared across the three growth groups using the non-parametric Kruskall-Wallis test followed by post hoc non-parametric sign tests with the Bonferroni’s correction. Categorical variables were analyzed with the chi-squared test or the Fisher’s exact test, as appropriate. Associations between changes in height z-scores at 12 months and other continuous variables were tested with the Spearman’s correlation coefficient (ρ). All calculations were performed with STATA/SE® for Windows, version 15 (StataCorp, College Station, TX, USA). Two-tailed *p* values <0.05 were considered statistically significant. 

## 3. Results

Table 1 shows the baseline characteristics of the entire study sample as well as separately for patients with DRE and GLUT1-DS. Thirty-four children (16 male and 18 female) were included in the study. DRE was diagnosed in 14 children, whereas the remaining 20 had GLUT1-DS. In the entire sample, the median age at diagnosis was 7.5 (4.0–10.0) years and the age at the beginning of KD ranged from 2 to 15 years (16 children were aged between 2 and 6 years, 10 between 7 and 10 years, and 8 between 11 and 17 years).

Patients with GLUT1-DS were diagnosed at an older age (median= 8.5 years; IQR = 5.0–10.0 years) than those with DRE (median = 4.0 years; IQR = 1.0–7.5 years; *p* = 0.041) and began dietary treatment earlier after diagnosis (median interval = 0.0 years; IQR = 0.0–1.0 years versus median = 4.7 years; IQR = 3.5–6.5 years, respectively, *p* < 0.001)—an expected finding being KD the only available treatment for GLUT1-DS.

All patients were in the pre-pubertal phase, with the exception of three subjects (1 with DRE and 2 with GLUT1-DS). There were significant differences in terms of anti-epileptic drugs (AEDs) and ambulation between the two groups. Specifically, all patients with GLUT1-DS were able to ambulate and none of them was taking AEDs. Ten patients with DRE were on multiple anti-epileptic drugs (AEDs) and their pharmacological treatment was not modified throughout the study. Moreover, five of them were unable to ambulate.

Table 2 shows the changes in growth status and body composition after 12 months of KD in the entire sample as well as separately for patients with DRE and GLUT1-DS. At baseline, there were no significant differences in median height z-scores between patients with DRE and GLUT1-DS (median = −0.4; IQR = 1.5−0.2 versus median = −0.7; IQR = −1.3−0.2, respectively, *p* = 0.675). No significant changes in all parameters emerged for both groups. Specifically, after 12 months of KD, the median height z-score did not change significantly from baseline both in patients with DRE (median = −0.4; IQR = −1.5–0.2 versus median = −0.4; IQR = −2.1−0.6; *p* > 0.90) and GLUT1-DS (median = −0.7; IQR = −1.3–0.2 versus median = −0.6; IQR = −1.4−0.3; *p* > 0.90). Based on these observations, all of the study patients were grouped together for analysis of growth patterns regardless of their underlying diagnosis. 

Group-I consisted of six children (2 male and 4 female) of whom 3 had GLUT1-DS. Three children were aged between 2 and 6 years and 3 between 7 and 10 years. One child was unable to ambulate. Group-T included 21 patients (11 male and 10 female) of whom 13 had GLUT1-DS. Eight children were aged between 2 and 6 years, 5 between 7 and 10 years, and 8 between 11 and 17 years. Three children were unable to ambulate. This group comprised three post-pubertal children. Finally, Group-D consisted of seven children (3 male and 4 female) of whom 4 had GLUT1-DS. Five children were aged between 2 and 6 years and 2 between 7 and 10 years. One child was unable to ambulate. The baseline characteristics of the three growth groups are summarized in Table 3. The three groups did not differ significantly in terms of clinical characteristics at baseline, the only exception being age at diagnosis (*p* = 0.028), which was lower in Group-D (median = 5 years; IQR = 3−6 years) than in Group-T (median = 9.0 years; IQR = 8−10 years; *p* = 0.041).

Table 4 shows the KD composition—in terms of energy and macronutrients—in three growth groups as well as the WHO recommendations for age ranges reflected in the study population.

A lower percentage of energy intake as carbohydrates was evident for Group-T (6.7%; IQR = 6.5−7.1%) compared with Group-I (4.4%; IQR 3.8−6.2%, *p* = 0.043).

The adherence to the prescribed dietary intakes was based on information provided by parents during control visits or through emails. Episodes of food refusal or incomplete meal consumption were reported for 11 of the 34 study patients, with the following distribution: one case out of 6 in Group-I, 7 cases out of 21 in Group-T, and 3 cases out of 7 in Group-D. No other adverse effects were observed throughout the study period.

Fasting β-OHB blood levels at 12 months did not differ significantly in the three study groups, being 1.8 mmol/L (IQR = 1.2–2.7 mmol/L) in Group-I, 2.4 mmol/L (IQR = 1.5–3.2 mmol/L) in Group-T, and 4.1 mmol/L (IQR = 1.9–6.1 mmol/L) in Group-D (*p* < 0.199). In the entire sample, a moderate inverse correlation was evident between fasting β-OHB blood levels at 12 months and the height z-score (ρ = −0.35; *p* = 0.04). 

Table 5 depicts the anthropometric and growth parameters (expressed as z-scores) observed in the three growth groups at baseline and after 12 months of KD. At baseline, there were no significant intergroup differences in terms of height, weight, or BMI z-scores. At 12 months, height (median = −1.4; IQR = −2.3–−0.6 versus median = 0.6; IQR = 0.3–0.9, respectively; *p* = 0.045) and growth velocity (median = −2.9; IQR = −3.6−-2.2 versus median = 2.2; IQR −0.2–4.2, respectively; *p* = 0.019) z-scores were significantly lower in Group-D than in Group-I.

After 12 months of KD, weight z-score was found to be more markedly impaired in Groups-D and -T than in Group-I—as shown by the following significant intergroup differences in ∆ weight z-scores: Group-I (median = 1.5; IQR = 0.5–2.5) versus Group-T (median = −0.3; IQR = −0.7–0.1; *p* = 0.003) or Group-D (median = −0.3; IQR = −0.7–−0.3; *p* = 0.008).

Fat mass percentage at baseline did not differ significantly in the three groups [Group-I (median = 32.5%; IQR = 30.1–34.3%), Group-T (median = 32.7%; IQR = 27.9−36.8%), and Group-D (median = 30.9%; IQR = 30.4–30.9%; *p* = 0.71)] and did not appreciably change at 12 months. 

In the entire sample at 12 months, there was a moderate positive correlation between height z-scores and weight z-scores (ρ = 0.580; *p* <0.001).

Detailed information on the three growth pattern subgroups are presented separately for patients with DRE and GLUT1-DS in Supplementary Tables S1–S3. 

## 4. Discussion

In this retrospective study, we sought to investigate the linear growth of children who remained on KD for 12 months. The results revealed that most of our patients (80%) maintained or even improved their growth at 12-month follow-up. Notably, this observation was consistent in children diagnosed with both DRE (of whom 11 out of 14 maintained or improved growth) and GLUT1-DS (among whom 16 out of 20 maintained or improved growth). Our findings are in accordance with those obtained in recent long-term follow-up studies. Wibisono et al. [26] reported their 10-year single-center experience with KD in Australia. Data from 33 children who had their height measured revealed that—during a 24-month period—height tracked along percentiles in 16 cases (48%). Increased and decreased height—by at least one standard deviation on percentile growth curves—was observed in seven (21%) and ten (30%) cases, respectively. Consequently, approximately 70% of their patients maintained or improved their linear growth pattern. In a prospective study, Lambrechts et al. [27] reported that 30% of their patients showed a growth deceleration after two years on KD. An investigation conducted in 50 Austrian children (mean age: 4.44 ± 3.55 years) with DRE, treated for 1.18 ± 1.06 years (minimum–maximum: 0.06−3.87 years) revealed that the frequency of growth retardation was as low as 6% [28].

Taken together, published evidence and our current data consistently indicate that growth retardation may occur in a minority of patients treated with the KD. From a clinical standpoint, it would be helpful to identify children at higher risk of poor growth before starting the KD. In an effort to address this issue, we compared the general characteristics of patients showing different growth patterns. However, Group-D was significantly different from other groups only in terms of age at diagnosis (which was found to be lower in this group compared to the Group-T). Because the interval between diagnosis and KD initiation did not show significant intergroup differences, the length of time during which the patient remained on a balanced diet cannot be a factor explaining the observed growth differences. Moreover, dietary prescriptions, supplementation patterns, and follow-up periods did not differ significantly in the three growth groups.

Potential causes of poor growth in children on the KD include inadequate intakes of energy and protein (compared with recommended values), the effects of the underlying diseases and treatments, acidosis/ketosis and related endocrine changes.

In a retrospective study conducted in 21 children (mean age: 7.9 ± 3.8 years), Williams et al. [10] reported a significant decrease in height and weight percentiles after two years on a classical KD. A prospective cohort study conducted in 237 children (age range: from 2 months to 9 years and 10 months) reported a small decrease in height z-scores in the first six months on a KD followed by larger changes at two years [29]. These studies were based on traditional ketogenic dietary regimens characterized by calorie restriction (75–85% of recommended daily energy) to achieve ketosis [30,31]. Therefore, their findings in relation to growth may have originated from inadequate calorie prescriptions. Calorie restriction for induction of ketosis is no longer used by most centers belonging to the International Ketogenic Diet Study Group [3]. In keeping with current guidelines, our study population started their KD regimen at full calorie (based on actual measures of energy expenditure which were subsequently fine-tuned at control visits)—an approach which may have prevented the decline in linear growth observed in earlier studies. 

To achieve a level of ketosis adequate for seizure control (in epilepsy) and for ketone use as an alternative fuel for brain metabolism (in GLUT1-DS), very low amounts of carbohydrates, adequate amounts of protein, and very high amounts of fat must be prescribed in a rigidly controlled ratio (ketogenic ratio)—resulting in a highly unbalanced diet compared to WHO recommendations for children and adolescents [21,22,23,24,25]. This kind of diet should be used only in presence of a medical condition that can be improved or cured by this regimen under a strict medical monitoring according to International Guidelines [3]. 

The maintenance of the ketogenic ratio limits the amount of proteins that can be included in the classical KD—although an amount of 0.8–1 g/kg body weight is generally provided. However, the amount of proteins needs to be carefully evaluated according to energy requirements in terms of protein-to-energy ratio [23]—an index of dietary quality particularly in the context of marginal food intake [23]. This topic was analyzed for the first time by Nation et al. [6], who demonstrated that a protein-to-energy ratio greater than 1.5 g protein/100 kcal is paramount to achieve satisfactory linear growth patterns in 35 children (age range: from 8 months to 16.5 years) treated with the KD for at least six months. All of the patients in the current study were prescribed a protein amount (expressed as percentage of total daily calories or as protein-to-energy ratio) in line with the WHO recommendations and the “appropriate ratio” according to Nation et al. [6]. We therefore believe that factors other than low energy or protein prescription should be considered to explain the decline in linear growth observed in some patients.

Growth patterns in children on KD may be influenced by the underlying disease as well as the presence of comorbidities and concomitant drug treatments. Patients with DRE may show suboptimal growth patterns even before the initiation of KD because of endocrine dysfunction or the use of multiple AEDs [32,33,34]. However, AEDs utilization did not show significant intergroup differences in our study. A direct comparison of our findings with those of other recent studies [9,13] is problematic owing to differences in the study population. Of the 22 children with growth deficit described by Spulber et al. [13], 21 had mental retardation, 14 autism spectrum disorder and nine were non-ambulatory; moreover, all of the children had been on long-term treatment with seven AEDs before KD implementation. Similarly, among the 15 children studied by Grouleau et al. [9] only those affected by cerebral palsy showed a −0.7 decline in height z-scores over a 15-month follow-up period. 

A single case of growth retardation related to GH deficiency has been reported in GLUT1-DS [15], making the exact incidence of poor growth difficult to estimate in this condition. 

In the current study, growth deficit was identified in only 20% of the study participants, without significant differences between children with DRE and GLUT1-DS. With the exception of one child in Group-D who was non-ambulatory, the main characteristic of our patients with delayed growth was a reduction in weight z-score, suggesting that periods of inadequate caloric intake may have occurred notwithstanding an adequate calorie prescription based on actual needs. A high-fat KD may be unpalatable for some subjects and can result in occasional food refusal, which was more frequently observed in Group-D (3 out of 7 children). However, a reduction in z-score was also observed in the Group-T. More interestingly, a trend toward higher ketone levels was evident in Group-D, albeit not significantly so (possibly because of the small sample size). 

A chronic systemic reduction of pH is known to impair growth [35] and KD may cause a persistent—albeit generally mild or clinically asymptomatic—metabolic acidosis. In this scenario, elevated levels of ketones induced by diets with high (4:1 and 3:1) ketogenic ratios may be another potential cause of poor growth. Accordingly, a negative correlation between height velocity and ketone levels has been reported for the first time by Peterson et al. [11] using a semi-quantitative urine dipstick test. Their results revealed that after 12 months on KD subjects with high urinary ketone levels (80–120 mg/dL) were characterized by lower height z-scores than those with moderate ketosis (<80 mg/dL). Spulber et al. [13] confirmed and expanded this observation by showing a negative correlation between blood β-OHB levels and both growth velocity and insulin growth factor-1 (IGF-1). IGF-1 is the key effector peptide for childhood linear growth in response to stimulation by growth hormone [36] and has been found to be decreased in starvation [37]. Fraser et al. [38] reported a 46% decrease in serum IGF-1 levels following seven days of a ketogenic diet that restricted CHO to less than 40 g/day while providing adequate energy and protein intake. It is thus possible that the starvation-like metabolic state induced by KD may reduce IGF-1 levels or decrease their bioavailability [9,13]. In a study conducted in 28 Swedish children, the Modified Atkins Diet (MAD—an alternative dietary treatment for epilepsy characterized by a low ketogenic ratio (0.8/1:1)—has been shown to induce lower levels of ketosis, which did not affect growth or IGF-1 levels at two years [39]. In line with this report [39], our dietary protocol routinely included oral citrates which—besides their affect against kidney stone formation—may also mitigate acidosis and its adverse effects on growth. In clinical practice, it is advisable to implement a close growth monitoring in all children with a high level of ketosis, aimed at eventually reducing the ketogenic ratio to lower values (albeit maintaining their compatibility with seizure symptoms control).

The caveats of our investigation include its retrospective design and the limited sample size. These limitations notwithstanding, our study describes for the first time the growth patterns of children with GLUT1-DS who had been treated with the KD. Importantly, most of our patients were able to maintain or improve their growth patterns. We speculate that our promising results may be ascribed to an accurate assessment of energy needs (obtained by repeated measurements of resting energy expenditure and a thorough assessment of daily physical activity levels), an adequate protein-to-energy ratio according to WHO recommendations, as well as to the continuing support offered to caregivers involved in the management of KD. Moreover, the routine supplementation of oral citrate may have mitigated the detrimental impact of chronic metabolic acidosis. However, more research is required to identify children at higher risk of growth delay. In addition, the potential role played by high ketone levels on altered IGF-1 signaling in influencing linear growth of children on KD needs further exploration. Under these circumstances, a more in-depth endocrine evaluation is advisable for all children who show evidence of growth retardation while on the KD. 

## Figures and Tables

**Table 1:** Baseline characteristic of the entire sample and of DRE and GLUT1-DS patients.

	ENTIRE SAMPLE	DRE	GLUT1-DS	*p*
	*n* = 34	*n* = 14	*n* = 20	
**Sex (male/female)**	16/18	7/7	9/11	>0.90
**Age at diagnosis (years)**	7.5 (4.0–10.0)	4.0 (1.0–7.5)	8.5 (5.0–10.0)	**0.041**
**Age at ketogenic diet initiation (years)**	8.0 (3.0–10.0)	4.0 (2.0–10.0)	9.0 (5.0–10.5)	0.310
**Interval between diagnosis and** **ketogenic diet initiation (years)**	0.5 (0–4.2)	4.7 (3.5–6.5)	0.0 (0.0–1.0)	**<0.001**
**Puberty (yes/no)**	3/34	1/13	2/18	>0.90
**Number of AEDs**				**<0.001**
**None**	21/34	1/14	20/20	
**1**	3/34	3/14	0/20	
**>1**	10/34	10/14	0/20	
**Ambulation**				**0.007**
**Yes**	29/34	9/14	20/20	
**No**	5/34	5/14	0/20	

Data are reported as counts, medians, and interquartile ranges, as appropriate. Abbreviation: DRE, drug resistant epilepsy; GLUT1-DS, glucose transporter type 1 deficiency syndrome; AEDs, anti-epileptic drugs; Significant *p* values are marked in bold (*p* < 0.05) for comparison between DRE and GLUT1-DS patients.

**Table 2:** Changes in growth status and body composition after 12 months on KD in the entire sample and in DRE and GLUT1-DS patients *.

	ENTIRE SAMPLE		DRE		GLUT1-DS	
	*n* = 34		*n* = 14		*n* = 20	
	Baseline	12 Months	Baseline	12 Months	Baseline	12 Months
**Height** **(cm)**	118.5 (95.0–143.0)	124.5 (104.0–148.0)	104.5 (92.5–137.5)	111.5 (99.0–139.0)	125.3 (107.0–148.0)	130.0 (113.0–153.5)
**Height z-score**	−0.6 (−1.4–0.2)	−0.6 (−1.8–0.5)	−0.4 (−1.5–0.2)	−0.4 (−2.1–0.6)	−0.7 (−1.3–0.2)	−0.6 (−1.4–0.3)
**Weight** **(kg)**	22.3 (15.0–37.7)	23.3 (17.7–42.1)	20.3 (11.6–35.6)	21.9 (13.6–34.4)	22.6 (16.8–40.1)	24.4 (18.9–46.5)
**Weight z-score**	−0.4 (−1.3–0.6)	−0.4 (−1.6–0.5)	−0.3 (−1.0–1.1)	−0.3 (−1.9–1.1)	−0.4 (−1.6–0.5)	−0.4 (−1.5–0.2)
**BMI** **(kg/m^2^)**	16.6 (15.3–19.4)	16.9 (15.0–18.0)	16.7 (15.0–19.4)	16.7 (14.6–18.0)	16.6 (15.5–18.6)	16.9 (15.0–18.1)
**BMI z-score**	0.2 (−1.0–0.9)	−0.1 (−0.8–1.0)	0.1 (−1.0–1.0)	−0.1 (−0.8–1.1)	0.2 (−0.9–0.8)	−0.1 (−0.9–1.0)
**FAT** **(% weight)**	32.4 (30.1–34.3)	29.9 (27.7–33.0)	32.9 (31.9–35.6)	30.8 (27.8–36.0)	30.9 (27.4–33.4)	29.2 (26.3–31.8)

Data are reported as medians and interquartile ranges. Abbreviation: DRE, drug resistant epilepsy; GLUT1-DS, glucose transporter type 1 deficiency syndrome; BMI, body mass index; FAT, fat free mass. * There were no significant differences between baseline and 12 months in each study group.

**Table 3:** Comparison of baseline characteristics in the three growth groups.

	Group-I (Increase)	Group-T (Track)	Group-D (Decrease)	*p*
	*n* = 6	*n* = 21	*n* = 7	
**Sex (male/female)**	2/4	11/10	3/4	
**Age at diagnosis (years)**	2.0 (2.0–7.0)	9.0 (8.0–10.0)	5.0 (3.0–6.0)	**0.028** **0.041** **
**Age at ketogenic diet initiation (years)**	5.5 (2.0–10.0)	9.0 (5.0–11.0)	5.0 (2.0–7.0)	0.084
**Interval between diagnosis and ketogenic diet initiation (years)**	1 (0–3)	0 (0–5)	0.5 (0–1)	0.727
**Puberty (yes/no)**	0/6	3/18	0/7	0.754
**Disease**				0.889
**Drug-resistant epilepsy**	3/6	8/21	3/7	
**GLUT1-DS**	3/6	13/21	4/7	
**Number of AEDs**				0.750
**None**	3/6	14/21	4/7	
**1**	1/6	1/21	1/7	
**>1**	2/6	6/21	2/7	
**Ambulation**				>0.90
**Yes**	5/6	18/21	6/7	
**No**	1/6	3/21	1/7	

Data are reported as counts, medians, and interquartile ranges, as appropriate. Abbreviation: GLUT1-DS, glucose transporter type 1 deficiency syndrome; AEDs, anti-epileptic drugs. Significant *p* values are marked in bold. The first *p* refers to three-group comparisons, being calculated from the non-parametric Kruskal–Wallis test. In the event of this P value being statistically significant (i.e., *p* < 0.05), the second *p* value indicates significant pairwise comparisons. Comparisons between ** Group-D and Group-T.

**Table 4:** Ketogenic diet composition—in terms of energy and macronutrients—in the three growth groups.

	WHO Recommendations	Group-I (Increase)	Group-T (Track)	Group-D (Decrease)	*p*
		*n* = 6	*n* = 21	*n* = 7	
**Energy intake (kcal/day)**	1088–2720 ^a^	1235.5 (788.0–1757.0)	1671.0 (1407.0–1805.0)	1282.0 (1150.0–1380.0)	0.085
**Energy intake (kcal/kg)**	51–82 ^a^	61.3 (51.2–81.2)	54.4 (41.5–66.7)	72.7 (62.5–95.8)	0.066
**Carbohydrate** **(% energy)**	55–75 ^b^	6.7 (6.5–7.1)	4.4 (3.8–6.2)	6.6 (4.0–9.9)	**0.028** 0.043 *
**Protein (% energy)**	3.7–5.5 ^c^	5.6 (5.4–7.1)	7.8 (6.5–8.5)	8.3 (6.5–8.7)	0.053
**Protein g/100 kcal**	0.9–1.4 ^c^	1.4 (1.4–1.8)	2.0 (1.6–2.1)	2.1 (1.6–2.2)	0.073
**Fat (% energy)**	25–35 ^d^	87.5 (87.1–87.5)	87.4 (87.2–87.5)	86.5 (79.1–87.4)	0.100
**Saturated fat** **(% energy)**	8 ^d^	21.2 (17.6–29.0)	31.3 (21.2–34.1)	25.7 (23.7–33.9)	0.452
**Monounsaturated fat (% energy)**	6–16 ^d§^	21.7 (20.4–26.4)	20.4 (16.1–24.5)	26.0 (24.5–29.9)	0.067
**Polyunsaturated fat (% energy)**	11 ^d^	5.5 (4.1–8.1)	5.7 (4.7–8.3)	8.6 (5.7–9.3)	0.368
**Cholesterol (mg/day)**	<300 ^d^	164.3 (107.3–217.2)	262.8 (137.1–347.5)	207.3 (179.0–289.8)	0.468
**Fiber (g/day)**	25 ^e^	4.8 (3.6–10.2)	6.8 (4.7–9.3)	7.2 (5.8–8.3)	0.751

Data are reported as medians and interquartile ranges. Significant *p* values are marked in bold. The first *p* refers to three-group comparisons, being calculated from the non-parametric Kruskal–Wallis test. In the event of this *p* value being statistically significant (i.e., *p* < 0.05), the second *p* value indicates significant pairwise comparisons. Comparisons between * Group-I and Group-T. *^a^* [21]; *^b^* [22]; *^c^* [23], *calculations were performed from values for protein and energy requirements (protein (g/kg) × 4)/ energy (kcal/kg). Safe protein: energy ratio for an individual calculated from protein and energy requirement values; ^d^* [24]; *^e^* [25]; *^§^* *Calculated as difference between Total fat – Saturated Fat Acids – Total Polyunsaturated fat.*

**Table 5:** Comparison between anthropometric and growth pattern characteristics (given as z-scores) of the three growth groups at baseline and after 12 months of ketogenic diet.

	Group-I (Increase)	Group-T (Track)	Group-D (Decrease)	*p*
	*n* = 6	*n* = 21	*n* = 7	
**BASELINE**				
**Height**	−0.4 (−1.1–0.3)	−0.6 (−1.5–0.2)	−0.8 (−1.0–0.2)	0.974
**Weight**	−0.9 (−1.8–0.1)	−0.1 (−1.1–1.1)	−0.6 (−1.3–0.3)	0.383
**BMI**	−0.9 (−1.4– −0.7)	0.3 (−0.5–1.3)	0.3 (−1.0–0.7)	0.256
**FAT (% weight)**	32.7 (27.9–36.8)	32.5 (30.1–34.3)	30.9 (30.4–30.9)	0.710
**12 MONTHS**				
**Height**	0.6 (0.3–0.9)	−0.5 (−1.6–0.3)	−1.4 (−2.3– −0.6)	**0.020** 0.045 ***
**Weight**	0.1 (−0.4–1.4)	−0.4 (−1.6–0.8)	−1.3 (−3.0– −0.1)	0.227
**BMI**	−0.4 (−0.7–1.2)	0.0 (−0.7–1.1)	0.3 (−1.6–0.6)	0.685
**FAT (% weight)**	34.1 (30.5–36.2)	28.4 (26.6–31.1)	31.4 (29.2–32.7)	0.110
**Growth velocity**	2.2 (−0.2–4.2)	−0.4 (−1.8–0.9)	−2.9 (−3.6– −2.2)	**0.002** 0.010 ** 0.019 ***
**∆ height**	0.9 (0.8–1.6)	0.0 (−0.1–0.1)	−1.1 (−1.2–0.3)	
**∆ weight**	1.5 (0.5–2.5)	−0.3 (−0.7–0.1)	−0.3 (−0.7– −0.3)	**0.002** 0.003 * 0.008 ***
**∆ BMI**	0.7 (0.1–1.3)	−0.3 (−0.6–0)	−0.1 (−0.5–0.1)	0.065
**∆ FAT (% weight)**	−2.4 (−3.6–1.4)	−1.3 (−5.4–0.3)	−0.2 (−0.7–0.0)	0.540

Data are reported as medians and interquartile ranges. Abbreviation: BMI, body mass index; FAT, fat free mass. Significant *p* values are marked in bold. The first *p* refers to three-group comparisons, being calculated from the non-parametric Kruskal–Wallis test. In the event of this *p* value being statistically significant (i.e., *p* < 0.05), the second *p* value indicates significant pairwise comparisons. Comparisons between * Group-I and Group-T, ** Group-D and Group-T, *** Group-D and Group-I.

## Supplementary Materials

The following are available online at https://www.mdpi.com/2072-6643/11/7/1442/s1, Table S1: Baseline characteristics of patients with DRE and GLUT1-DS presented separately and in relation to the three different patterns of growth, Table S2: Ketogenic diet composition—in terms of energy and macronutrients—of patients with DRE and GLUT1-DS presented separately, Table S3: Anthropometric and growth pattern characteristics—(given as z-scores unless otherwise indicated)—observed at baseline and after 12 months of ketogenic diet in patients with DRE and GLUT1-DS presented separately.
