# Supplementary material for: Impact of the Ketogenic Diet on Linear Growth in Children: A Single-Center Retrospective Analysis of 34 Cases
Source: Nutrients. 2019 Jun 26;11(7):1442. doi: 10.3390/nu11071442 (PMC6683244; doi:10.3390/nu11071442)
Supplement: Supplementary file 1 [file nutrients-11-01442-s001.pdf]

Supplementary Materials

Table S1. Baseline characteristics of patients with DRE and GLUT1-DS presented separately and in relation to the three different patterns of growth

	**DRE**			**GLU1-DS**		
	**Group-I (Increase)**	**Group-T (Track)**	**Group-D (Decrease)**	**Group-I (Increase)**	**Group-T (Track)**	**Group-D (Decrease)**
	**n =3**	**n =8**	**n =3**	**n =3**	**n =13**	**n =4**
**Sex (males/females)**	1/2	5/3	1/2	1/2	6/7	2/2
**Age at diagnosis (years)**	4.5 (2.0-7.0)	4.5 (0.8-9.0)	3.5 (1.0-6.0)	2.0 (1.0-10.0)	9.0 (8.0-11.0)	5.0 (4.0-6.0)
**Age at ketogenic diet initiation (years)**	9.0 (2.0-10.0)	4.0 (2.5-12.5)	3.0 (2.0-10.0)	2.0 (2.0-10.0)	10.0 (9.0-11.0)	5.5 (3.5-6.5)
**Interval between diagnosis and ketogenic diet initiation (years)**	4.5 (2.0-7.0)	4.5 (0.8-9.0)	3.5 (1.0-6.0)	2.0 (1.0-10.0)	9.0 (8.0-11.0)	5.0 (4.0-6.0)
**Puberty (yes/no)**	0/3	1/7	0/3	0/3	2/11	0/4
**Number of AEDs**						
**None**	0/3	1/8	0/3	3/3	13/13	4/4
**1**	1/3	1/8	1/3			
**>1**	2/3	6/8	2/3			
**Ambulation**						
**Yes**	2/3	5/8	2/3	3/3	13/13	4/4
**No**	1/3	3/8	1/3			

Table S2. Ketogenic diet composition – in terms of energy and macronutrients – of patients with DRE and GLUT1-DS presented separately

	DRE				GLUT1-DS			
	**Entire sample**	**Group-I (Increase)**	**Group-T (Track)**	**Group-D (Decrease)**	**Entire sample**	**Group-I (Increase)**	**Group-T (Track)**	**Group-D (Decrease)**
	**n=14**	**N=3**	**N=8**	**N=3**	**n=20**	**n =3**	**n =13**	**n =4**
**Energy intake (kcal/day)**	1504.5 (1150.0-1757.0)	1602.0 (788.0-1757.0)	1539.0 (1183.5-1770.5)	1150.0 (987.0-1806.0)	1545.5 (1318.5-1852.0)	869.0 (748.0-1899.0)	1684.0 (1541.0-2004.0)	1318.5 (1240.5-1367.5)
**Energy intake (kcal/kg)**	58.3 (51.2-66.7)	57.1 (51.2-65.6)	57.0 (47.4-64.8)	85.8 (50.7-95.8)	60.2 (45.7-76.9)	81.2 (42.0-97.1)	53.1 (41.5-70.1)	72.0 (66.9-89.4)
**Carbohydrate (% energy)**	4.8 (4.0-7.0)	6.8 (5.1-7.1)	4.3 (3.1-5.4)	8.8 (4.0-21.8)	5.7 (3.8-6.8)	6.5 (6.5-9.5)	5.1 (3.8-6.2)	6.1 (4.5-8.2)
**Protein (% energy)**	6.9 (6.4-8.1)	5.5 (5.3-7.1)	7.3 (6.4-8.0)	8.3 (6.5-8.4)	7.9 (5.9-8.6)	5.8 (5.4-8.1)	8.0 (6.8-8.6)	7.8 (6.4-9.2)
**Protein (g)/100 kcal**	1.7 (1.5-2.0)	1.4 (1.3-1.8)	1.8 (1.5-2.0)	2.1 (1.6-2.1)	1.9 (1.5-2.2)	1.4 (1.4-2.0)	2.0 (1.7-2.1)	2.0 (1.6-2.3)
**Fat (% energy)**	87.5 (87.4-87.8)	87.5 (87.4-89.6)	87.5 (87.5-87.8)	82.0 (71.0-87.5)	87.2 (85.8-87.4)	87.3 (87.2-87.4)	87.1 (82.2-87.5)	86.8 (82.8-87.3)
**Saturated fat (% energy)**	22.2 (17.6-31.5)	17.6 (16.8-24.7)	19.8 (17.8-31.6)	30.3 (25.7-35.0)	31.1 (23.6-35.0)	29.0 (17.8-41.7)	32.4 (24.7-36.0)	23.8 (21.8-28.9)
**Monounsaturated fat (% energy)**	22.9 (16.0-27.0)	21.6 (15.6-21.8)	20.1 (15.2-25.7)	29.9 (25.9-36.0)	22.7 (18.4-26.4)	26.4 (20.4-29.3)	20.4 (17.7-23.7)	25.2 (21.8-27.7)
**Polyunsaturated fat (% energy)**	9.4 (4.8-15.2)	9.0 (3.6-11.5)	8.9 (4.8-16.9)	14.2 (4.8-17.5)	11.5 (8.3-15.1)	6.7 (3.3-30.6)	11.6 (8.3-17.0)	11.1 (9.4-12.3)
**Cholesterol (mg/day)**	177.5 (107.3-241.8)	184.1 (107.3-217.2)	143.9 (105.0-238.1)	241.8 (128.8-369.5)	276.5 (179.0-370.0)	144.5 (59.9-370.0)	299.2 (228.5-441.9)	203.3 (189.1-248.6)
**Fiber (g/day)**	6.7 (4.5-9.1)	6.0 (3.6-10.2)	5.8 (3.3-8.2)	8.3 (7.2-12.8)	6.7 (4.2-9.5)	3.6 (3.2-11.3)	7.3 (5.2-9.9)	5.9 (5.0-6.8)

Table S3. Anthropometric and growth pattern characteristics – (given as z-scores unless otherwise indicated) – observed at baseline and after 12 months of ketogenic diet in patients with DRE and GLUT1-DS presented separately

	**Entire sample**	**Group-I (Increase)**	**Group-T (Track)**	**Group-D (Decrease)**	**Entire sample**	**Group-I (Increase)**	**Group-T (Track)**	**Group-D (Decrease)**
	**N=14**	**N=3**	**N=8**	**N=3**	**n=20**	**n =3**	**n =13**	**n =4**
BASELINE								
**Height**	104.5 (92.5-137.5)	130.0 (92.0-140.5)	104.5 (93.8-137.8)	95.0 (83.0-137.5)	125.3 (107.0-148.0)	82.6 (74.5-142.0)	143.0 (124.0-150.0)	108.0 (97.8-109.8)
**Weight**	20.3 (11.6-35.6)	23.8 (12.7-31.4)	20.3 (13.1-40.3)	11.6 (11.5-35.6)	22.6 (16.8-40.1)	10.4 (7.7-45.2)	34.5 (21.1-42.5)	17.8 (14.8-19.8)
**BMI**	16.7 (15.0-19.4)	15.0 (14.1-15.9)	18.1 (16.0-21.4)	16.7 (13.3-18.8)	16.6 (15.5-18.6)	15.5 (13.9-22.4)	16.7 (15.6-19.8)	16.6 (15.3-16.7)
**FAT (% weight)**	32.9 (31.9-35.6)	32.9 (16.4-36.8)	32.9 (32.2-34.6)	33.6 (30.9-36.3)	30.9 (27.4-33.4)	32.4 (27.9-39.0)	31.4 (27.0-34.3)	30.4 (23.7-30.9)
12 MONTHS								
**Height**	111.5 (99.0-139.0)	135.2 (102.5-146.5)	111.5 (100.8-140.0)	99.0 (88.0-139.0)	130.0 (113.0-153.5)	96.0 (82.0-155.0)	146.0 (128.0-154.0)	113.0 (101.5-114.0)
**Weight**	21.9 (13.6-34.4)	28.4 (18.5-34.4)	21.9 (15.6-36.7)	12.7 (10.8-37.5)	24.4 (18.9-46.5)	14.2 (10.1-55.7)	33.0 (22.5-49.3)	19.0 (16.1-21.2)
**BMI**	16.7 (14.6-18.0)	16.0 (15.5-18.0)	17.6 (15.4-18.6)	13.9 (13.0-19.4)	16.9 (15.0-18.1)	15.4 (15.0-23.2)	17.2 (15.0-18.6)	16.4 (14.9-17.2)
**FAT (% weight)**	30.8 (27.8-36.0)	34.3 (30.5-38.2)	28.4 (27.7-33.0)	34.4 (32.7-36.0)	29.2 (26.3-31.8)	34.1 (28.8-36.2)	28.4 (24.2-30.7)	30.1 (26.4-31.4)
**Growth velocity**	-1.1 (-1.9-0.9)	0.5 (-1.1-2.2)	-0.6 (-1.8-0.9)	-3.6 (-5.0- -2.2)	-0.4 (-2.6-1.0)	4.2 (-0.2-11.7)	-0.2 (-1.7-0.7)	-2.9 (-3.4- -2.2)
**Δ Height**	-0.0 (-0.3-0.2)	0.8 (0.8-2.9)	-0.0 (-0.1-0.1)	-1.2 (-1.3- -1.1)	-0.0 (-0.3-0.1)	0.9 (0.5-1.6)	0.0 (-0.1-0.1)	-0.5 (-0.8--0.3)
**Δ Weight**	-0.4 (-0.7-0.5)	1.2 (0.5-2.9)	-0.5 (-0.9-0.0)	-0.7 (-2.3- -0.3)	-0.2 (-0.5-0.1)	1.9 (0.2-2.5)	-0.3 (-0.6-0.1)	-0.3 (-0.4--0.1)
**Δ BMI**	-0.1 (-0.8-0.4)	1.1 (0.0-2.2)	-0.3 (-0.8-0.2)	-0.1 (-1.9- -0.1)	-0.1 (-0.5-0.5)	0.3 (-0.1-1.3)	-0.4 (-0.5-0.0)	-0.0 (-0.3-0.4)
**Δ FAT (% weight)**	-1.3 (-5.8-1.4)	-0.5 (-2.4-1.4)	-5.4 (-8.3-4.1)	-0.3 (-0.3- -0.3)	-1.2 (-3.7- -0.0)	-3.6 (-4.9-8.3)	-1.2 (-3.8- -0.5)	-0.2 (-1.2-0.2)
